# Sexuality-based stigma and access to care: intersecting perspectives between healthcare providers and men who have sex with men in HIV care centres in Senegal

**DOI:** 10.1093/heapol/czac010

**Published:** 2022-02-11

**Authors:** Albert Gautier Ndione, Fanny Procureur, Jean-Noël Senne, Francesca Cornaglia, Khady Gueye, Cheikh Tidiane Ndour, Aurélia Lépine

**Affiliations:** Faculté des Lettres et Sciences Humaines, Université Cheikh Anta Diop Dakar, BP 5005 Dakar-fann, Dakar 10700, Senegal; Institute for Global Health, University College London, 30 Guilford Street, London WC1N 1EH, UK; French Institute of Research for Development, Paris-Saclay, University Paris-Sud, 3 Rue Joliot Curie 2e ét, 91190 Gif-sur-Yvette, France; School of Economics and Finance, Queen Mary University of London, Mile End Rd, Bethnal Green, London E1 4LJ, UK; Ministry of Health and Social Action of Senegal Fann Résidence, Rue Aimé Césaire, Dakar, Senegal; Ministry of Health and Social Action of Senegal Fann Résidence, Rue Aimé Césaire, Dakar, Senegal; Institute for Global Health, University College London, 30 Guilford Street, London WC1N 1EH, UK

**Keywords:** Stigma, MSM, HIV, Senegal, key populations

## Abstract

Men who have sex with men (MSM) in Senegal face a challenging socio-legal context, marked by homophobia and the illegality of homosexuality. In addition, human immunodeficiency virus (HIV) prevalence among MSM is 27.6%, 46 times greater than the one in the general population (0.5%). Nevertheless, access to healthcare by MSM may be hampered by stigmatizing attitudes from health facility staff (medical and non-medical). This article describes the health facility staff/MSM relationship and analyses its effects on access to healthcare by MSM. The data used were collected through a field survey based on observations and qualitative interviews conducted in 2019 and 2020 with 16 MSM, 1 non-governmental organization (NGO) staff and 9 healthcare providers in Dakar (the capital city) and Mbour (secondary city on the West Coast) hospitals. The data were subject to a thematic analysis assisted by the ATLAS software. The relationship between MSM and healthcare providers is ambiguous. On the one hand, healthcare providers are torn between their professional duty to treat MSM and the cost of being stigmatized by other colleagues. Therefore, they often limit their empathy with MSM within the hospital context. On the other hand, MSM, trusting in the confidentiality of healthcare providers, feel safe in the care pathway. However, we identify the following stigmatizing factors limiting access to care include (1) fear of meeting a relative, (2) difficult relationships with non-medical support staff (mainly security guards), (3) HIV status disclosure and (4) potential conflicts with other MSM. This study is unique as it includes non-medical staff in its respondents. It shows that hospitals are divided into several areas, based on the stigma perceived by MSM. It is important to map out MSM’s care trajectories and spaces and to identify all types of staff working within them, including non-medical staff, and enrol them in stigma reduction interventions.

**Key messages**

- Access to healthcare by men who have sex with men (MSM) in Senegal is hampered by severe stigma.
- MSM, trusting in the confidentiality of medical staff, feel safe in their care relationship.
- Stigmatizing factors limiting access to care include (1) fear of meeting a relative, (2) difficult relationships with non-medical staff (security guards, cleaners), (3) HIV status disclosure and (4) potential conflicts with other MSM.
- It is important to map out all staff working around MSM care to enrol them in stigma reduction interventions.

## Introduction

Senegal is a country with a concentrated human immunodeficiency virus (HIV) epidemic estimated at 0.3% in the general population (UNAIDS, 2020), but with higher rates amongst key populations (Mukandavire *et al.*, 2018). The prevalence of HIV is estimated at 6.6% among female sex workers, 5.2% among injecting drug users, 2% among prisoners and as high as 27.6% among men who have sex with men (MSM) (CNLS, 2019; Mukandavire *et al.*, 2018). Among MSM, the HIV prevalence varies strongly across regions; Dakar (49.6%), Diourbel (34.8%) and Mbour (32.7%) are the most affected localities (CNLS, 2019). According to the Senegalese Ministry of Health, although most probably a lower bound, the estimated 18+ MSM population size for the Dakar region is around 1840 (Minsitry of Health, 2016).

Studies on MSM in Senegal show that homosexual and bisexual practices are complex and that their analysis is necessary to better understand and prevent HIV vulnerability among MSM in Senegal (Larmarange, 2009). The roles of violence and stigma on access to specific care and their impact on HIV prevention and treatment efforts (Dramé *et al.*, 2013; Ireland, 2013; Foley, 2010) have also been described in the literature.

At the societal level, the homophobic context manifests itself through the qualification of ‘unnatural’ practices to define homosexual sex acts and that are perceived as a religious ban in a country where Muslims make up 95% of the population (Broqua, 2016; N’diaye, 2012). These ‘unnatural’ acts are criminalized by Article 319 of the Penal Code, which punishes ‘unnatural sexual practices between persons of the same sex’ with a maximal 5-year jail sentence and a fine of CFAF 1 500 000 (euros 1000). Literature shows that the intentional politicization of homophobia under religious pressure has served as a reassertion of national integrity in the face of Western domination and as a tool of electoral support (Angotti *et al.*, 2019; Bertolt and Masse 2019; M’Baye and Muhonja 2019). The media has also been a great contributor in depicting same-sex relationships as a decadent menace to society (Mbaye, 2021). Within this context, homophobic attitudes towards MSM are also reported within families and communities (Broqua, 2012; Ferguson, 2017). For instance, a national survey measuring the prevalence of stigma among people who live with HIV (PLWHIV) showed that MSM are highly stigmatized, with 28.3% of them who had to change their place of residence due to intense stigma (RNP+, 2012). This also shows the pressure of intersecting stigma (Friedland *et al.*, 2018) MSM have to face in many public spheres.

These different levels of vulnerability and layers of stigma raise the issue of the potential barriers in access to care and services for MSM. However, the relationship between health staff and MSM is poorly documented in Senegal. The anthropological literature on medical health staff describes healthcare workers as social actors with a set of beliefs that may differ from biomedical norms and that are conveyed in acts of care (Faye, 2009; Lyons *et al.*, 2017; Jaffré and Sardan, 2003). In addition, non-medical staff are frequent in healthcare facilities in Senegal; they include guards, vendors and cleaners. So far, there is no evidence of the perception of non-medical staff regarding MSM and on their role in healthcare access. The issue of stigma by health staff has strong public health implications since if stigma acts as a barrier in the demand for care by MSM, it can affect not only their access to prevention services but also their access and adherence to antiretroviral drugs, thus favouring the transmission of the epidemic. Yet, the issue of access to care of MSM has important public health implications since they are seen as a ‘bridge population’, with the potential to spread the epidemic to the general population. Indeed, it has been documented that a large proportion of MSM are married to a woman to conceal their homosexuality or reduce family pressures to marry (Larmarange, 2009).

In order to analyse the MSM/medical staff relationship, we refer to the theoretical concept of stigma. The term ‘stigma’, since its use in Goffman and Goffman (1963) has undergone a historical and conceptual evolution based on its nature, sources and consequences (Link and Phelan 2001). It is defined as the characterization of an individual or group that is perceived to deviate from the norms of the group (Goffman and Goffman 1963) and implies a distinction between ‘us’ and ‘them’ (Devine *et al.*, 1999; Morone, 1997).

In the field of HIV, many papers have defined conceptual frameworks related to stigma (Earnshaw and Chaudoir 2009; Holzemer *et al.*, 2007; Parker and Aggleton 2003). Stigma against people living with HIV, identified as part of the ‘third phase of the epidemic’ (Chambers *et al.*, 2015), manifests itself in health facilities through the denial of treatment, disclosure of HIV status and humiliating attitudes (Schuster *et al.*, 2005; Sears *et al.*, 2012). In this paper, we will use Scrambler’s differentiation between ‘enacted stigma’ referring to episodes of discrimination against a person for a specific attribute and ‘felt or internalized stigma’, relating to the shame associated with the stigmatizing attribute or the fear of encountering enacted stigma (Scambler, 2004).

Studies have shown that stigma against PLWHIV have negative effects on mental health (Fife and Wright 2000; Sandelowski *et al.*, 2004) taking the form of anxiety (Gonzalez *et al.*, 2009; Ivanova *et al.*, 2012; Wagner *et al.*, 2010), depression (Lee *et al.*, 2002; Peltzer and Ramlagan 2011; Rueda *et al.*, 2012; Secor *et al.*, 2015; Ulanja *et al.*, 2019), suicidal thoughts (Rodriguez-Hart *et al.*, 2017; Stahlman *et al.*, 2016), life dissatisfaction (Greeff *et al.*, 2010) and poor quality of life (Holzemer *et al.*, 2009; Vyavaharkar *et al.*, 2012).

Among MSM themselves, self-stigma has been explained through the concept of ‘internalized homophobia’ and is subject to influences from socio-political and individual sources (Berg *et al.*, 2017). It is another key factor in understanding health–seeking-related behaviours of MSM. Indeed, internalized homophobia has been analysed as a source of problems within MSM relationships (Frost and Meyer 2009) that can be a barrier to the use of HIV prevention services (Ross *et al.*, 2008; Santos *et al.*, 2013).

Using qualitative evidence from in-depth interviews with 16 MSM, 1 non-governmental organization (NGO) staff and 9 health staff (including 4 non-medical staff) in the two cities of Dakar and Mbour, this article aims to address two main research questions. First, it aims to document the perceptions involved in the care relationship between MSM, medical, and especially non-medical staff, which has not been done in other studies. More specifically, the paper highlights the different forms that stigma can take in this relationship as well as their perceived origins according to both MSM and health staff (medical and non-medical). Second, the paper documents the effect of stigma on access and quality of healthcare received by MSM. Finally, this paper also highlights other public spaces in which MSM are the most stigmatized, which contributes to a better understanding of their persecution in Senegalese society.

The paper is organized as follows. ‘Methodology’ Section presents the methodology and data collection procedures used for this research. Results are presented in ‘Results’ Section and discussion is done in Section ‘Discussion’ and is followed by ‘Conclusion’.

## Methodology

The data come from a field survey carried out in Senegal between 2019 and 2020. The survey sites included two hospitals in two different cities. The first site was located in a tertiary university hospital in Dakar, and the second site was located in a tertiary hospital in Mbour. In terms of layout, the Dakar hospital had a dedicated HIV care centre for MSM only, and the Mbour hospital had a HIV care centre for all ‘at risk populations’ such as sex workers, people who inject drugs and MSM. The hospitals involved in the survey are multi-purpose hospitals with several departments, each housed in a separate building. They are independent of each other even if they remain connected through interpersonal relationships between caregivers or through referral of patients. Thus, healthcare staff generally know the activities of each department. However, the main entrance is a neutral zone and no one knows where patients are going, except possibly from guards who recognize regular patients as they often act as receptionists.

Dakar, the capital city of Senegal, is characterized by its large population size and numerous HIV care centres, while Mbour, at the coastal zone, is characterized by its touristic attractiveness often associated with significant sex tourism (Salomon, 2009) and the low presence of HIV care centres.

We included four categories of participants in our sample: medical staff (*n* = 5), non-medical staff working in and around the hospital (*n* = 4), HIV-positive MSM (*n* = 11) and HIV-negative MSM (*n* = 5), and one NGO staff (*n* = 1). A total of 16 MSM were interviewed. Criteria for inclusion were (1) to be older than 18 years and (2) having sex with at least another man at the time of the survey. The sample was further stratified using age, HIV status (positive or negative) and use or non-use of care services. ‘Non-use of care services’ refers to study participants who were not using services at the time of interview but who had used services in the past and no longer wanted to use them or who had indirectly interacted with the hospital (through hearing stories of close friends or accompanying close friends) and consequently decided not to use those services. We want to note that our sample is not representative of the MSM population in terms of HIV status, as participants were recruited from a hospital setting where prevalence is likely to be higher than in the general population.

Contact with MSM was facilitated by a peer outreach mediator^1^ from a partner NGO, who helped us identify various profiles of MSM. Before putting us in contact with MSM, the mediator approached them either by telephone or by going to certain parts of town where they usually gather to recruit MSM who responded to the selection criteria. He then presented the study as an exploration of relationships between health staff and MSM and introduced them to the researcher (name, profession, experience, place of work and place of residence). We opted for this method as this population is very hidden and hard to reach, and thus, we needed an intermediary. Medical and non-medical staff were recruited on the basis of their involvement with MSM caregiving, from reception to care and from social assistance to treatment. Staff were recruited through a request to the hospital head, who was briefed on the need for diversity in their characteristics (age, sex, level of education and position).

Data collection was carried out in three phases. The first phase consisted of semi-structured interviews with 10 MSMs and 6 medical staff and observations of care sites in Dakar and Mbour. In the second phase, interviews were transcribed, cleaned and analysed. The third phase included semi-directive interviews with five additional medical staff and a focus group with six MSM to collect additional information that did not appear in previous phases and to validate the information analysed in phase two.

For health staff, interview topics included career paths, experience in providing HIV treatment, experience in treating key populations, perceptions of MSM, information/training about MSM and difficulties in the care relationship with MSM. For MSM, topics included care trajectories, preferred places to seek care, financial resources to seek care, quality of healthcare, attitudes of medical staff, difficulties experienced in the care relationship, expectations in treatment and perception of stigma within the hospital and other institutions (e.g. legal, religious and care provided by non-governmental organizations). Most interviews with MSM were done in Wolof and the rest was done in French by our main researcher who is Senegalese.

Interviews were conducted during off-peak hours for medical staff and at the time of their choice for MSM. Almost all medical staff were interviewed at their workplace, except for one doctor who was interviewed at his/her home. Interviews with MSM were conducted at the location of their choice, which was outside their neighbourhood (restaurant, friend’s home, health centre/hospital).

All participants were informed of the objectives and outcomes of the research. An information sheet was provided to each participant. Each section was explained in French or Wolof. Health staff were asked to choose a pseudonym and to replace any name they would mention in the interviews by ‘X’. This process of anonymization was also used during transcription. A supplementary consent form was read and signed by each research participant. Participation in the survey was unpaid. However, travel expenses of all participants were reimbursed up to 5000 CFA francs (euro 7.5). The duration of the interviews varied between 40 and 70 minutes.

All recorded interviews were transcribed, cleaned and re-anonymized if needed be. Qualitative data were then subjected to mixed thematic analysis using the Atlas software. This thematic analysis was both deductive and inductive. More precisely, the analysis was based on topics from the interview guides, but also any other recurring themes were also added to the analysis. We also identified any links between general themes and sub-themes. Field notes including relevant details such as non-verbal communication and choice of place of interview were also used to complement analyses. Selected quotes were translated from French to English for the purpose of this paper.

## Results

Among the 16 MSM participating in the survey, 11 were HIV positive, 8 resided in Dakar and 8 in Mbour. They were aged between 22 and 45 years and were mostly single (*n* = 14). Only two were married, including one who had two children. Most of them had completed primary (*n* = 6) and secondary (*n* = 5) education. Only two had not attended school. In terms of occupation, the sample of MSM included one student, one teacher, three mediators and two who were a hotel employee and a tailor. All other MSM (*n* = 9) were in urban informal employment.

The sample of medical staff included six women and four men. There were two doctors, three nurses, two cleaners, two guards and one mediator. Their ages varied between 30 and 56 years with professional experience varying between 1 and 16 years. Their levels of education included primary (*n* = 2), middle (*n* = 3), secondary (*n* = 3) and higher education (*n* = 2).

Detailed information on the characteristics of health providers and MSM surveyed are presented in Supplementary Appendix 1.

### Being MSM: perceptions of ‘causes’, categories and risks

Using various terms such as homosexuals or *goordjigen*^2^ MSM are often described by Senegalese society as religious deviants. Categories and risks associated with homosexuality by the respondents reveal an uneasy balance between a strong cultural and religious influence and medical references.

*Religion prohibits all three (drug use, sex work and homosexuality), but I find that often the latter two are worse. When you talk about homosexuality, they tell you it’s haram* (Fatou, medical staff).

The term *haram* means a ‘prohibited, unlawful behaviour (…) forbidden by law, statute or sharia’ (Ajami, 2008). The reference to the dominant Muslim religion is also made by some MSM who refer to their status as a *nattu* (burden or hardship) imposed by a divine will^3^ and thus not chosen, as opposed to others who assume their sexual orientation as a life choice or a human right.

#### Perceptions and beliefs on homosexuality

Often based on the perception that no one is ‘born homosexual’, health staff put forward several reasons why someone ‘becomes’ MSM.

*I could mention three things: money. They think it’s a lobby, that if you enter their group, they will give you money (…) The other is that it’s fashionable and they want it (…) For others it’s their childhood that catches up with them, because they had suffered from unnatural sex acts as children and they got used to it* (Diafra, medical staff).

These comments encompass the ‘causes’ of homosexuality as they are generally perceived by medical staff, for whom the environment in which MSM grew up was a determining factor in their practices. As Dieynaba, medical staff, puts it:

*Sometimes you see a guy who grows up with women only, and he does everything like them until he adopts all their practices without even realizing it*.

The financial reason is also frequently mentioned and linked to the belief that there is a ‘gay lobby’ that encourages, protects and supports MSM. Particularly in the locality of Mbour, the pursuit of money is also associated with sexual transactions with tourists. In this locality, some health workers additionally identified two categories of MSM: one referring to people who have ‘become’ MSM through socialization, and male sex workers.

*They don’t all claim to be MSM: there are some who claim to be ‘guy-fuckers’ (…) or sex workers: they are on the street and looking for money, there are some who are looking for a stable couple* (Diafra, medical staff).

Other specific beliefs on homosexuality were noted among non-medical staff, like security guards and cleaners who work in health facilities.

*The system can be responsible in some way; tests are done, and when some people are HIV positive and find themselves marginalised, they can be drawn into that MSM environment. (…) They are attracted and integrated by MSM. Some say ‘I can no longer live with a woman at the risk of infecting her’ (…) and therefore they prefer to go with a man whom they cannot infect* (Balla, security guard).

This shows a lack of understanding of the link between being HIV-positive and being MSM among this category of workers. However, this quote interestingly points to a certain understanding of a particular marginalization that occurs after HIV diagnosis.

#### Perceptions of categories of MSM

By using the oppositions ‘men and women’ and ‘passive and active’, the perception of health staff and non-medical staff echoes with MSM’s own categorizations, who often use the terms *ibbi* and *yoos*^4^ to distinguish between those who practice the ‘active’ and ‘passive’ sex or ‘those who play the role of the man and those who play the role of the woman’ during sexual intercourse.

Contrastingly, medical staff also perceive MSM to be divided into two other categories, but which are based on their marital status and disclosure of their MSM status, as if MSM were choosing between these categories. However, this difference only reflects varying social pressures.

*I have seen some who call themselves homosexuals but who have wives and tell you they want children* (Fatou, medical staff).

*Absolutely, there are those who hide and those who don’t* (Dieynaba, medical staff).

Marriage was also mentioned by MSM as a social pressure that highlights the rejection of homosexuality by the Senegalese society. When a family becomes aware or suspects that one of its members is a MSM, it will suggest him to marry a woman to hide a sexual orientation that will not be accepted.

#### Risks associated with homosexuality

The description of perceived health risks associated with MSM status mostly relates to HIV and sexually transmitted infections (STIs), as reported by medical staff. Infectious diseases are always highlighted, while HIV is systematically mentioned by all respondents. Indeed, MSM are perceived as high-risk of being infected with HIV, even if, in reality, any individual MSM is still more likely to be HIV-negative than HIV-infected.

*I think about certain diseases (…) HIV, STIs and clinical conditions such as anal widening* (Moussa, medical staff).

*There are even some who have fistulas and come to get a dressing (…) When they have unprotected sex, they are exposed to diseases such as hepatitis, HIV and others* (Astou, medical staff).

*Infections, STIs, hepatitis, anal fracture* (Nafi, medical staff).

Yet, in addition to the biomedical risks put forward in their professional practices, some medical staff also perceive MSM as a threat to society.

*Because they are most often rejected by society, so when they do their things, it’s in secret (…). It is precisely in these circumstances that the transmission of diseases will occur, and they return to the population, to the family; as a result, there is a threat to society* (Diafra, medical staff).

*Two men can’t have children, it changes society to some extent and can even spoil it* (Balla, security guard).

*In Islam, it is recommended that they are killed (…) Because this can lead to the apocalypse according to the Koran; and a whole population should not pay for the mistakes of a few people* (Dieynaba, medical staff).

The idea of a threat associated with MSM takes on three dimensions according to both medical and non-medical staff. MSM are perceived as a potential source of diseases in the population and a threat to the reproduction of the human species. Finally, referring to religion and the example of Lot^5^, MSM could potentially cause the wrath of God leading to the end of the world.

### Caregiving relationship between medical staff and MSM

#### MSM perceptions of hospital staff

MSM mostly refer to medical staff as ‘open’, ‘understanding’ and ‘welcoming’.

*Open, welcoming, and also patient (…) because the doctors are very open, they talk with the patient until you talk about what you want to hide* (P5).

*The doctor is someone with whom you can share your illness confidentially, thirdly if he knows you have HIV he will tell you what to do* (P8).

*Understanding. There are always a few medical staff who have been trained and despite their rank, they are willing to receive us and listen to us; so yes, they are understanding on that aspect* (P9).

The generic term ‘doctor’ is the one used to refer to all medical staff wearing a white coat including doctors, nurses, laboratory staff and social workers,^6^ as opposed to non-medical staff (e.g. security guards in particular).

*Social workers who support you even if it’s not in the context of the illness, they call you, they talk to you, which was rare at that time. They consider you as their children* (P5).

#### Caring for MSM: a relationship perceived as risky by medical staff

MSM reported that some medical staff did not have an empathetic attitude from the beginning because of socio-religious norms, but that this empathy emerged over the course of the care relationship.

*There are some doctors who don’t want to touch them and conjure up religious excuses* (Astou, medical staff).

Some medical staff eventually come to terms with their initial shock and enter a long-term care relationship that they describe as difficult because of the fear of being identified as MSM ‘sympathizers’. Indeed, providing care to MSM means carrying the stigma of ‘supporting’ their cause, which health providers see as a potential threat to their reputation. The other risk identified by health staff working with MSM is the stigma by colleagues in other services.

*When a man, who has all the physical attributes, suddenly acts like a woman, in a health facility open to everyone, where there are elderly people and notables, it is complicated. You feel ashamed because that is where you work. Even if it is for professional reasons, people might think that we are like them because we support them* (Fatou, medical staff).

*When people see you joking around with MSM, especially elderly, they say you look like a homosexual* (Astou, medical staff).

#### Stigma and access to care for MSM

The hospital is a public place where MSM are confronted with various people from whom they may suffer stigma. Indeed, non-medical staff were often mentioned by MSM as the main category of people from which they were experiencing stigma and discrimination.

*There is a MSM who tested positive (for HIV), so he had to go to the hospital for a confirmation test (…). But when he left, he had a problem with the security guards when he said he wanted to meet a social worker (…). He said their gaze was awkward, plus they were talking about him as soon as they saw him come in* (peer mediator).

*Sometimes you meet a security guard you know, but within the hospital he acts as if he does not know you. But whether it’s the doctors, the social workers, the nursing staff, frankly we don’t have any problem* (P5).

Therefore, the hospital comprises a trajectory in which MSM suffer both enacted stigma taking the form of verbal violence and negative attitudes from non-medical staff and felt or internalized stigma, as they anticipate feeling shame when seen in the hospital. Indeed, in addition to the poor treatment from security guards and cleaners, MSM also fear to meet close relatives among health workers and other patients.

*We arrived at the hospital with a friend, we found two MSM there. Two days later I heard that one of them is sick (with HIV), I believe this rumour because I saw him that day at the hospital. That’s why a lot of MSM don’t want to go to hospitals* (P4).

*One of my friends found out his HIV status during a screening, but because his aunt is in the hospital, he doesn’t want to risk being seen* (P10).

Peer mediators assigned to care sites thus play an important role in this context. Indeed, they facilitate access and adherence to care. Better known as on-site mediators, they act as an interface between MSM, medical staff and non-medical staff within health facilities.

*We enter the hospital to take his ARVs, if he needs a check-up I speak with the social worker so that he can come when there are less people* (peer mediator).

Moreover, HIV-positive MSM face a risk of double stigmatization based on their sexual orientation but also their HIV status, which they do not want relatives to find out.

One interviewee also mentioned the stigma from their peers:

*Even if you lose weight, they will start to say you have the disease. Plus, they will prevent you from seeing other MSM by asking them to avoid you* (P12).

The risk of marginalization based on HIV status not only causes MSM to refuse testing and hide their status but also leads some of them to refuse to visit the hospital to avoid meeting other MSM who may become aware of their HIV status.

*One day I was called for a HIV screening, and I was told to take 5 people with me. When we came, we realised that there were around 50 people there. If I had known, I would not have come, because we are hiding* (peer mediator).

This quote also points out the unintended consequences of efforts to increase testing among at-risk populations.

Our qualitative data also provide some evidence that internal factors specific to the organization of the MSM community are also crucial for access to care. The MSM community is far from being homogenous and at least two groups of MSM, namely non-sex workers versus sex workers, had some stigmatizing behaviours towards the other group. MSM also expressed the fear of meeting other MSM with whom they do not get along around the hospital.

*There are some MSM that I don’t greet; I’m about to enter a shop when I see them to avoid passing them, or to take a taxi or to leave. (…) Because he turns out to be a showy personality and discretion is a quality that God himself appreciates* (P3).

Several types of conflicts (fighting, rivalry and jealousy) were reported among MSM and have repercussions in access to healthcare facilities. Some MSM say they avoid hospitals, if they have to meet ‘flags’ (who openly disclose sexual orientation), who risk to unintentionally ‘out’ them when interacting with them. Mediators often say that these internal conflicts within MSM communities make it difficult for them to bring MSM together for HIV prevention activities (screening, talks, etc.).

In summary, there are various stigmatizing factors that prevent MSM from seeking care: (1) the fear of meeting someone they know, (2) the difficult relationship with non-medical staff, (3) places dedicated to key populations including MSM, (4) their HIV status disclosure and (5) intra-MSM conflicts. Our data shows that HIV-positive MSM are the most affected by these because they face double stigmatization. Indeed, even if our analysis does not directly demonstrate that HIV-infected respondents report more stigmatizing incidents than those who were HIV-negative, our respondents did mention that they would use the belief that a person was infected with HIV as a ‘weapon’ to prejudice MSM because they know it will cause them ostracization.

### Perceived safe places for MSM

In a scenario in which MSM were asked to rank three pictures—(1) hospital, (2) police station and (3) mosque or church—from the most discriminating to the least discriminating place, MSM revealed various forms of stigma experienced within these institutions. The majority of our respondents ranked the hospital the least discriminating place. This was in high contrast with police stations and religious institutions, which were systematically described as places where they felt the most uncomfortable.

#### Discrimination in the religious institution and in the police force

MSM refer to the relationship between religion and homosexuality as a complex subject that is differently interpreted by religious authorities, as religious representatives are often torn between neutrality and reprobation.

*Most of the time it is the media which force religious people to react. There is a great marabout* *^7^*  *who made a statement saying that it is not written anywhere in the Koran that an MSM should not be buried. Only God is judge* (P9).

*I can say that it was the mosques that primarily stigmatize MSM. Yesterday there was an MSM who died, we wanted to bury him in a Muslim cemetery but people did not accept it* (P10).

One of the major discriminations perceived by MSM is the refusal to be buried in a religious cemetery, of which several examples are cited, such as the MSM who was excavated after burial^8^. MSM point out a sensational press, which uses homosexuality as a ‘hot topic’ to attract readers’ attention, always reviving the debate towards prohibition and reprobation. MSM also denounce religious sermons that focus solely on the *haram* nature of homosexuality, which pushes MSM to think that their health is worthless. This has of course many implications for HIV prevention and treatment.

*Since I’m already in it, I’m already cursed, so it doesn’t matter what I do’* (P14).

The police are also reported as a place of high stigma where being labelled a homosexual systematically leads to mistreatment and abuse, whatever the reason of the dispute.

*When you are a MSM you can be hurt (but) it is enough for the person to say that you are an MSM and immediately nothing else matters. You are isolated, you don’t even have the right to speak* (P3).

The attitude of the police towards MSM is reported to be the source of several problems First, it ‘allows’ the population to call them ‘*goordjigen*’ and to attack them freely. Second, because of this stigma by the police, MSM say they do not dare to go and file a complaint for fear that their MSM status will be prejudicial to them. Attitudes that aim to label someone as homosexual to get out of a dispute are also often used.

MSM also report that being publicly mistreated by the police not only reveals their MSM status but can also encourage other persons, especially in prison, to abuse them.

However, from the point of view of non-medical staff interviewed in this study, legal sanctions against MSMs are currently not as severe as they should be.

*The state has the most responsibility in this (…) because it can prohibit or allow many things. If I were a friend of the President, I would tell him to issue a decree, so that all those who behave as such suffer sanctions so heavy that they would be dissuasive* (Amy, cleaner).

*We could start by going to see the imams and all the other media, to sensitize the entire population and make people understand that we want this to disappear, apply visible sanctions to all those caught in the act, and that everyone witnesses it; maybe at that point it will decrease* (Zara, cleaner).

#### The hospital, a place like no other for MSM?

MSM all associate safety with the hospital because of the trusted and understanding medical staff with whom they interact directly.

*They (social workers) are my friends because the way they welcome us is something else. They don’t even see us as MSM, they see us as any other person* (P5).

*I will always go there because they already know me, they know who I am, but they also know my status. I tell myself that if I change hospital, other people will know who I really am and it will backfire* (P10).

In contrast to the religious and penal institutions, the hospital is a place where MSM can assert their status with less fear in the context of care relationships. They are reassured by the nature of the place and by people who are trained and ready to welcome and treat them as human beings. However, MSM, like medical staff, believe that this sympathetic relationship is mostly based on professional obligations and is not necessarily synonymous of tolerance or support outside of the hospital context.

*We can smile and all that, but I know that it’s because of the work that binds us together because normally we wouldn’t be so cordial to each other. It is imposed on them because of their work, because they need us and we need them, but it is not of their own free will* (P9).

*It is in the context of work and it is said that working is submission to God. So, in this spirit, I do what I have to do without making distinctions between categories of people* (Fatou, medical staff).

The care of MSM is considered as a professional duty to persons who have the right to health and as a form of devotion to God that does not conflict with any religious principles. However, these relationships cannot exist outside of the hospital.

*There are doctors who avoid them, but I don’t do it because I tell myself that it’s in the context of work: when I leave the hospital I have no affinity with them (…). I can be familiar with them, talk and joke with them. (…) But outside of here, we don’t see each other and we don’t even cross paths* (Astou, medical staff).

Even if medical staff may have negative perceptions, they are willing to enter a professional relationship. This explains why the hospital is a perceived safe place for MSM in their interactions with the dedicated medical staff.

## Discussion

The description of the care relationship by both MSM and medical staff shows the ambiguity of their rapport. Medical staff, under the banner of professional duty, agree to enter an empathic relationship with MSM exclusively in the health facility. MSM, trusting the confidentiality of the medical staff, feel a sense of security in the healthcare facilities that they do not experience in other institutions. This result echoes the one from a study conducted in Malawi in which medical staff were willing to provide appropriate healthcare because it was perceived as their professional responsibility (Kapanda *et al.*, 2019). Other studies on HIV care amongst MSM in Africa have established that the stigma associated with HIV plays an important role in the difficulties encountered by MSM in their care trajectory (Maleke *et al.*, 2019; Pilgrim *et al.*, 2019). Our study also highlights a link between stigma, poor mental health and sexual risk behaviours in the light of religious pressures, which mirrors a recent Nigeria study (Rodriguez-Hart *et al.*, 2017).

We show that the hospital is mostly seen as a safe place where MSM feel free to talk about their sexual orientation and their HIV status. This positive experience in the care setting has also been described as facilitating the access to care (Ogunbajo *et al.*, 2018). Our findings are yet different from most African studies that usually show that the stigma through denial of care or poor treatment (Grosso *et al.*, 2019; Lyons *et al.*, 2017), the feeling that health workers do not care (Kushwaha *et al.*, 2017) and the breach of confidentiality (Gamariel *et al.*, 2020) are the main barriers to MSM’s access to care.

Our study results have several policy implications. Firstly, the study identifies the attitude of non-medical staff, although not strong in numbers, as a main barrier to healthcare of MSM, reinforcing the necessity to involve non-medical staff in training to improve healthcare access of MSM, as another study has shown in Senegal (Lyons *et al.*, 2017). In addition, the comparison between experiences of stigma within different institutions also shows the potential for engaging with police officers and religious leaders in order to reduce the homophobia in Senegalese society and to enforce the human rights of MSM. It is in this light that part of our research team worked on a documentary that accompanies this paper, illustrating MSM’s daily struggles (Lepine *et al.*, 2021).

The hospital seems to be divided into several spaces. On the one hand, common areas where there is a risk of meeting a relative, or being stigmatized by other patients or security guards, can be considered as a place of high stigma. On the other hand, services dedicated to MSM care with face-to-face relationships with health staff are perceived as low-stigma places. This was the case for the Dakar hospital, which had a designated area for MSM only. This strengthened discretion for MSM, as opposed to the Mbour hospital in which all ‘at-risk populations’ were grouped in the same care area and thus could potentially draw more attention to MSM. It is important to map out these care spaces and trajectories to identify all staff, especially non-medical, in need of training as well as hospital layout improvement to enhance discretion and confidentiality.

Secondly, beyond the aspects related to health facilities, reasons for discouraging access to care also show that the conflictual relationships between MSM constitute a strong barrier to healthcare. Conflicts between different groups of MSM, which originate outside the health care structures, can affect the use of health services, so it is crucial to minimise these conflicts through mediators. Indeed, the work of mediators facilitates access and adherence to care, and to antiretroviral treatment. However, given the numerous conflicts among the MSM population, future research should study further how to best select and train mediators in order to reduce tensions within MSM groups and improve their capacity overall.

Lastly, HIV-positive MSM face strong intersectional stigma (Lyons *et al.*, 2020; Turan *et al.*, 2019) as they, beyond the fear of being seen by a relative in health facilities, also suffer from marginalization within the MSM community. It prevents their access to care, particularly in the context of activities involving other MSM, and more efforts should be deployed in order to reduce stigma among PLWIH MSM in Senegal.

## Limitations

Our study includes some limitations. Firstly, we were only able to interview only 16 MSM, given our time and budget constraints. We acknowledge that this constitutes a weakness in our study in terms of representativeness and data saturation. Indeed, it is impossible to claim that our sample is representative of the MSM population. However, we made sure that the sample was very varied in terms of age, marital status and HIV status. Secondly, given the hidden and hard-to-reach character of our target population, participant selection was only made possible through the intervention of peer outreach mediators. This could have slightly biased participant selection as mediators might have known some of the participants, but we believe that it was the best option to approach and recruit this population. Finally, we acknowledge that the number of non-medical staff interviewed is a very small sample; however, this population (security guards, cleaners) also represents a very small portion of staff present in health facilities and we believe that the cited quotes reflect prevailing perceptions among non-medical staff.

## Supplementary Material

czac010_Supp

## Data Availability

The data underlying this article cannot be shared publicly due to the sensitive nature of the data and potential identifiers which would not protect the privacy of individuals that participated in the study. The data will be shared on reasonable request to the corresponding author.

## Endnotes

1. Peer mediators are people from MSM communities, selected and trained by local NGOs, who help facilitate the link and rapport between MSM and caregivers at the health facility level.

2. In the Wolof society, the term most often used to name the MSM is the word *goordjigen*, which literally translates to ‘male-female’ or ‘transgender’. This word implies the idea of a man who is very similar to a woman, to the point of identifying with them (Hayes, 2011; Niang *et al.*, 2002). It is used in Senegal to capture both non-cisgender and homosexual behaviours and appearances (Bertolt and Masse 2019).

3. By qualifying their MSM status as a *nattu*, respondents comply with a popular perception in Senegal according to which each believer is subjected to a ‘test’ that God inflicts on him and from which he must pass.

4. Among themselves, MSM prefer the terms *Ibbi* and *Yoos*. ‘the Ibbi is a person more inclined to adopt feminine mannerisms and less dominant behaviours in sexual interactions. (…) The Yoos is usually the penetrating partner during sexual intercourse, but does not consider himself to be homosexual’. The terms are also explained more simply as receptive vs insertive (Niang *et al.*, 2002, p. 9).

5. Lot is mentioned in the Bible and the Koran, he went to the city of Sodom to remind those practising homosexuality that they broke the divine law. Determined to continue with homosexual practices, the wrath of God fell on their city and killed them all. In the Koran, the first mention of this story is in chapter 7, verses 80–84.

6. Social workers are part of NGOs just as peer outreach mediators but have followed specific training and are state social workers. They usually have more senior roles in NGOs and supervise peer mediators.

7. A Muslim holy man or hermit. They are the religious leaders from Senegal’s Sufi brotherhoods and thus an incredibly important influence on the lives of Senegalese people.

8. The famous 2008 viral video of the corpse of a man who was dug up and dragged out of a cemetery by several people in Thies, Senegal (https://www.jeuneafrique.com/160338/societe/le-corps-d-un-homosexuel-pr-sum-exhum-thi-s/).
