# Supplementary material for: Sexuality-based stigma and access to care: intersecting perspectives between healthcare providers and men who have sex with men in HIV care centres in Senegal
Source: Health Policy Plan. 2022 Feb 11;37(5):587–96. doi: 10.1093/heapol/czac010 (PMC9113117; doi:10.1093/heapol/czac010)
Supplement: czac010_Supp [file czac010_supp.zip › Appendix.docx]

# Appendix

Appendix 1: Characteristics of interviewees

1. Health staff

| **Pseudonym** | **Age** | **Sex** | **Education level** | **Town** | **Function** |
| --- | --- | --- | --- | --- | --- |
| Balla | 54 | M | Middle school | Mbour | Security guard |
| Fatou | 30 | F | Secondary | Mbour | Assistant nurse |
| Diafra | 35 | M | Tertiary | Mbour | Doctor |
| Dieynaba | 47 | F | Secondary | Mbour | Nurse |
| Astou | 50 | F | Secondary | Mbour | Assistant nurse |
| Moussa | 56 | M | Tertiary | Dakar | Doctor |
| Ibrahim | 46 | M | Middle school | Dakar | Security guard |
| Amy | 32 | F | Primary | Dakar | Cleaner |
| Zara | 29 | F | Primary | Dakar | Cleaner |
| Nafi | 42 | F | Middle school | Dakar | HIV Mediator |

b) MSM

| **Pseudonym** | **Age** | **Education level** | **Marital status** | **Function** | **HIV status** |
| --- | --- | --- | --- | --- | --- |
| P1 | 29 | Secondary | Single | Hotel industry | positive |
| P2 | 35 | None | Married | Factory worker | positive |
| P3 | 32 | Primary | Single | Restaurant waiter | positive |
| P4 | 23 | Secondary | Single | Student | positive |
| P5 | 25 | Secondary | Single | Salesman | positive |
| P6 | 42 | Primary | Single | Stylist | negative |
| P7 | 24 | Primary | Single | Dancer | negative |
| P8 | 30 | Primary | Single | Plumber | negative |
| P9 | 30 | Tertiary | Single | Teacher | negative |
| P10 | 31 | Primary | Single | Dancer | positive |
| P11 | 32 | Secondary | Single | Mediator | positive |
| P12 | 22 | Middle school | Single | Mediator | positive |
| P13 | 37 | Middle school | Single | Mediator | positive |
| P14 | 35 | Secondary | Married | Decorator | negative |
| P15 | 36 | None | Single | Painter | positive |
| P16 | 23 | Primary | Single | Tailor | positive |
